# Tracheocutaneous Fistula Resolved by Pentadecapeptide BPC 157 Therapy Through the NO-System—Triple NO-Agent Approach in Rats

**DOI:** 10.3390/ph19010145

**Published:** 2026-01-14

**Authors:** Goran Madzarac, Tomislav Becejac, Toni Penovic, Dominik Drazenovic, Lucija Kralj, Marta Popović Dolic, Suncana Sikiric, Lidija Beketic Oreskovic, Ivana Oreskovic, Sanja Strbe, Ana Maria Tubikanec, Mihovil Penavic, Hrvoje Vranes, Ivan Krezic, Mario Kordic, Antun Koprivanac, Tinka Vidovic, Josipa Vlainic, Dinko Stancic Rokotov, Alenka Boban Blagaic, Sven Seiwerth, Anita Skrtic, Predrag Sikiric

**Affiliations:** 1 Department of Pharmacology, School of Medicine, University of Zagreb, 10000 Zagreb, Croatiatonipenovic@gmail.com (T.P.); kordic.m@gmail.com (M.K.);; 2 Department of Pathology, School of Medicine, University of Zagreb, 10000 Zagreb, Croatia; 3 Laboratory for Advanced Genomics, Division of Molecular Medicine, Institute Ruder Boskovic, 10000 Zagreb, Croatia

**Keywords:** tracheocutaneous fistula, BPC 157, L-arginine, L-NAME, L-NAME+L-arginine, rats

## Abstract

**Background/Objectives:** This 7-day rat tracheocutaneous fistula study considered the not-studied issues of tracheocutaneous fistula course, wound healing, and fistula in the NO-system relations. Therefore, we focused on fistulas’ severe course, tracheocutaneous fistula → air leak → compensatory diaphragmatic/abdominal “heaving”, NO-system failed relations, and therapy resolution. Stable gastric pentadecapeptide BPC 157 was proposed. **Methods**: Tracheocutaneous fistula rats received daily medication (/kg), alone or combined, BPC 157 therapy (10 µg, 10 ng, in drinking water or intraperitoneally) along with a triple NO-agent approach (L-NAME 5 mg, L-arginine 100 mg, and L-NAME+L-arginine, intraperitoneally). **Results:** Tracheocutaneous fistulas occurred as specific and NO-system-related as follows: NO system: blockade (L-NAME-aggravation) over-activity (L-arginine-amelioration) or immobilization (L-NAME+L-arginine oppose each other’s effects). Controls presented severe clinical signs of respiratory distress, failed healing, skin and tracheal defects, a not-healed and open, macro/microscopically, and fistulous tract that was well-formed and wide, tracheal shrinking below the fistula, and clinically, open-mouth breathing, “heaving abdomen”, cyanosis (bluish snout, ears, extremities), abundant secretion through the fistula, and weight loss. Fistula tissue NO level decreased, and the malondialdehyde (MDA) level increased. The BPC 157 therapy (both application routes) resulted in rapid recovery. Healing of defects (skin and trachea) and fistula closure, macro/microscopically, corresponded with clinical findings, avoiding observable clinical signs of dyspnea, reducing weight loss, and avoiding any sign of “heaving abdomen”. BPC 157-treated rats displayed regular breathing movements without observable signs of respiratory distress. Finally, when combined, BPC 157 therapy upgrades L-arginine amelioration, abolishes L-NAME-induced worsening, and restores full healing after NO immobilization (L-NAME+L-arginine). BPC 157 counteracted increase in NO level and counteracted increase in MDA level. **Conclusions:** Thus, first, acting systemically, BPC 157 reverses tracheocutaneous fistula course in rats. It acts through the NO system.

## 1. Introduction

This study of tracheocutaneous fistula in rats [1] considered the not-studied problem of tracheocutaneous fistula healing [2,3,4,5], wound healing, and fistula issues as an NO-system problem [2,3,4,5]. For this considerable theoretical (NO-system–tracheocutaneous fistula relation undetermined) and practical gap (i.e., no available pharmacotherapy), we proposed a solution. This was stable gastric pentadecapeptide BPC 157, a wound healing and cytoprotective therapy [6,7,8,9,10], particularly for fistula healing [11,12], closely interacting with and modulating the NO system [6,7,13,14,15], and a triple NO-agent approach. The triple NO-agent approach maintains the NO-system function as a whole, examining the L-NAME (NO-system blockade), L-arginine (NO-system over-activity), and L-NAME+L-arginine (NO-system immobilization) applied in the same study [6,7,13,14,15]. There, based on the NO agents’ effects [16,17,18], BPC 157 has been shown to counteract NO inhibition (L-NAME) (i.e., hypertension, pro-coagulant effect [16,18]) and NO overactivity (L-arginine) (i.e., hypotension, anti-coagulant effect [16,18]). In addition, BPC 157 induces the release of NO by itself, resistant to L-NAME, opposing the L-arginine-induced NO release [16,17]. Mechanistically, activation of the VEGFR2-Akt-eNOS signaling pathway appeared, without the need for other known ligands or shear stress [19,20,21]. Controlling the vasomotor tone, both smooth muscle and endothelium is maintained through the activation of the Src-Caveolin-1-eNOS pathway [19,20,21].

Therefore, all relevant NO states (i.e., NO system as a whole) [6,7,13,14,15] combined with tracheocutaneous fistula healing define the tracheocutaneous fistula healing course. In contrast, the common conventional approach (the application of a single NO agent) would be more limited. The L-NAME application alone (thereby, NO blockade only) is commonly used in the literature (see, i.e., [22,23,24,25,26,27]). On the other hand, BPC 157 therapy would resolve the fistula in all relevant NO states [11,12].

As mentioned, the NO system is crucial for vascular tone regulation, angiogenesis, epithelial integrity, and inflammation [6,7], all key components of successful tissue repair, and thereby tracheocutaneous fistula as well.

Currently, the basic research on tracheocutaneous fistula pharmacotherapy has involved only two studies: one in humans (a case report) [28] and the other in animals [1]. In rats, a tracheal defect of 1.5 mm diameter was a prototype [1], while this study used a markedly larger tracheal defect. In a broader fistula context (pharyngocutaneous [29,30], tracheoesophageal [31], gastrointestinal [32], and bronchopleural [33,34,35]), various pharmacotherapy attempts (stem cells from human exfoliated deciduous teeth [1], ozone [29], botulinum neurotoxin A [30], mesenchymal stem cells [31], fibrin glue [32], ethanol [33,34], and silver nitrate [28,35]) occurred with local deposition or injection. In addition, considering the peptide application, there is a notable lack of evidence that exogenous administration of single recombinant growth factors alone (without carrier addition) directly induces fistula closure (note a few reports on platelet-derived growth factor (PDGF) [36] and fibroblast growth factor (bFGF) [37]). There are local platelet-rich plasma (PRP) and concentrated growth factor (CGF), which deliver autologous combinations of PDGF, vascular endothelial growth factor (VEGF), epidermal growth factor (EGF), and transforming growth factor-β (TGF-β) within a fibrin scaffold [38,39,40,41,42]. In contrast, BPC 157 acts alone (without carrier addition), as a particular advantage for peptide therapy (i.e., BPC 157 is native and stable in human gastric juice for more than 24 h, acting as a cytoprotection mediator), it can be easily administered and acts systemically, even via the per-oral route [6,7,8,9,10,11,12,13,14,15].

Thus, there is still no reliable pharmacological therapy capable of promoting spontaneous or assisted tracheocutaneous fistula closure.

On the other hand, this property distinguishes BPC 157 from exogenous growth factors and locally applied biological therapies, which generally require carriers, scaffolds, or direct tissue application to achieve efficacy. BPC 157 therapy exhibits a particular point in wound healing [8,9]. There is the simultaneous healing of different tissues, providing fistula healing [11,12], both external [43,44,45] and internal [46,47,48,49]. Notably, in the general cytoprotection concept [50,51,52,53,54,55,56,57,58,59,60,61], these pleiotropic beneficial effects align with its role (i.e., cytoprotection mediator) in implementation. As conceptually defined [50,51,52,53,54,55,56,57,58,59,60,61], cell protection appears to be an innate activity of a cytoprotective agent [6,7,8,9,10,11,12,13,14,15]. This also includes, after the long term, the reversal of already formed fistula that persisted for a considerable time [48]. This capability could be, with the application of NO agents, L-NAME (NO inhibition), L-arginine (NO over-stimulation), and L-NAME and L-arginine given together (L-NAME+L-arginine, NO system immobilization), especially applied to resolving all complexities of tracheocutaneous fistula healing.

Likewise, for tracheocutaneous fistula therapy, BPC 157 was expected to exhibit high therapeutic effectiveness. Notably, this can be expected with the activity consistently observed within the 10 µg–10 ng/kg dose range [6,7,8,9,10,11,12,13,14,15]. Toxicology studies have shown a favorable safety profile; a harmless limit test at doses up to 2 g/kg (i.v. or i.g.) in mice, without adverse effects, and with no lethal dose (LD1) was achieved (for details, see [6]). This point was confirmed in other studies as well [62,63,64]. Clinically, it has been much less applied than in animal research [6,7,8,9,10,11,12,13,14,15]. BPC 157 has been successfully evaluated in a phase II trial for ulcerative colitis, where it proved effective and well-tolerated, without adverse effects [65,66]. More recently, it has shown successful application in the management of knee pain and interstitial cystitis [67,68]. Thus, we used a protocol (triple NO-agent approach) commonly applied in BPC 157 studies and, now, after tracheocutaneous fistula formation. BPC 157 was given in drinking water, using its abovementioned advantage as a cytoprotective mediator [6,7,8,9,10,11,12,13,14,15]. Alternatively, BPC 157 was given using a daily intraperitoneal regimen, as described before [6,7,8,9,10,11,12,13,14,15]. This was along with the once-daily intraperitoneal administrations of L-NAME, L-arginine, and L-NAME+L-arginine [6,7,8,9,10,11,12,13,14,15], a protocol regularly used in our studies [6,7,8,9,10,11,12,13,14,15], including fistula studies (esophagocutaneous, duodenocutaneous, and colocutaneous fistula [43,45,46]).

In addition, the study included an investigation of the NO level and MDA level in tissue. Namely, as reviewed as a part of the particular modulatory effect of BPC 157 on the NO-system’s functions [6], the NO level in tissue, increased or decreased, was regularly normalized through BPC 157 administration, along with a decrease in the increased MDA-level.

This should provide an accurate novel relation of tracheocutaneous fistula with the NO system as a whole that could be essential for the whole course. Furthermore, the clinical course of tracheocutaneous fistula—including open-mouth breathing, cyanosis, and a characteristic “heaving abdomen”—reflects the severe clinical manifestations of respiratory distress. These signs are consistent with behavioral patterns described in experimental models of respiratory distress, where abdominal muscle recruitment represents a compensatory response to increased airway resistance or impaired airway integrity [69,70,71,72,73,74,75,76,77]. Accordingly, animal deterioration, dyspnea-like behavior, tracheal shrinking, and failure of fistula closure, regularly presented, worsened (tracheocutaneous fistula → air leak → increased inspiratory effort → abdominal recruitment → progressive distress) or recovered would reflect distinct NO-system states: blockade (L-NAME), overstimulation (L-arginine), and immobilization (L-NAME+L-arginine). The consistent effect of BPC 157 on fistula lesions and NO-system modulation, achieved via both oral and intraperitoneal administration, supports the robustness of these findings within the limits of clinical observation.

## 2. Results

### 2.1. Tracheocutaneous Fistula

**Figure 1:**
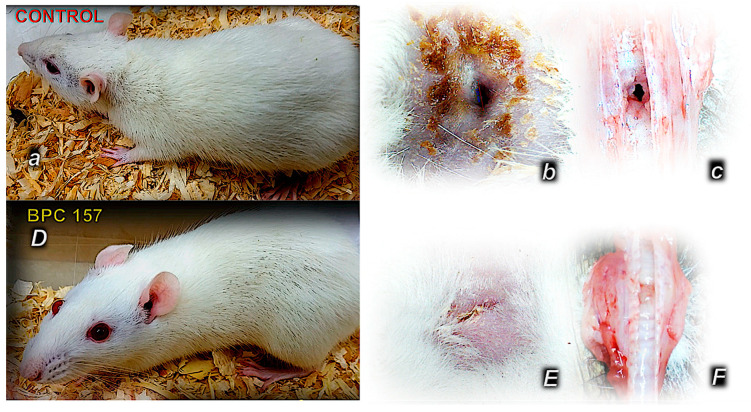
Rat clinical presentation and gross skin and tracheal defects after tracheocutaneous fistula creation in controls (normal letters, **a**–**c**) and BPC 157 (capital letters, **D**–**F**). Shortly after the formation of the tracheocutaneous fistula, rats exhibited marked clinical signs of respiratory distress due to air leakage, including pronounced abdominal excursions (“heaving abdomen”), flared nostrils, open-mouth breathing, and cyanosis (bluish coloration of the snout, ears, and extremities). There was abundant secretion through the fistula and markedly reduced activity. Both the skin and tracheal defects remained open without healing (**a**–**c**), accompanied by tracheal narrowing below the fistula, and continuous weight loss was observed. These were largely avoided in BPC 157-treated rats, where the skin and tracheal defects were closed (**D**–**F**), and the weight loss was reduced.

Regularly, the control rats presented with failed tracheocutaneous fistula healing from the beginning to the end of the experiments. Soon after tracheocutaneous fistula formation, due to air leakage, rats presented severe clinical signs of respiratory distress, pronounced abdominal excursions or a “heaving abdomen” during respiration, flared nostrils, and open-mouth breathing, cyanosis (bluish snout, ears, extremities), abundant secretion through the fistula, and reduced activity (Figure 1, Table 1 and Table 2).

**Figure 2:**
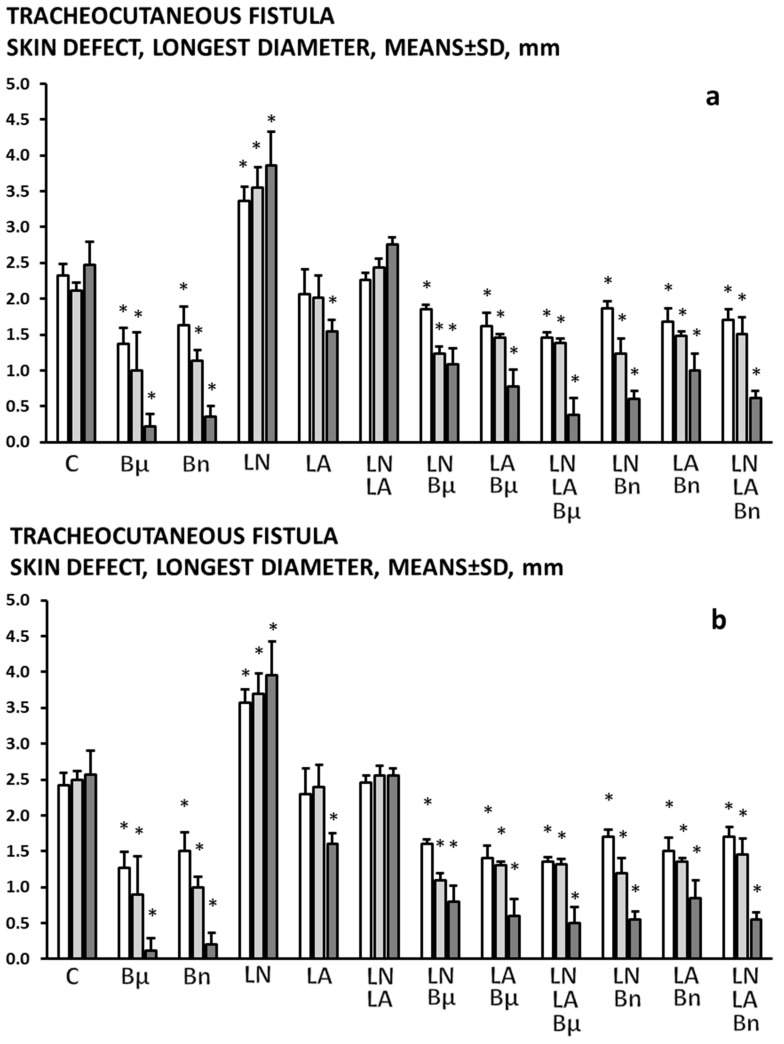
Tracheocutaneous fistula skin defect, longest diameter, means ± SD, mm, at post-operative day 3 (white bars), 5 (light gray bars), and 7 (dark gray bars). BPC 157 was given per-orally, in drinking water (10 μg/kg (Bμ), 10 ng/kg (Bn), i.e., 0.16 μg/mL, 0.16 ng/mL, 12 mL/rat/day) until sacrifice on the third, fifth, and seventh postoperative day (**a**) or, alternatively, 10 μg/kg, 10 ng/kg intraperitoneally, with the first application at 30 min after surgery and the last at 24 h before sacrifice (**b**). Likewise, with the first application at 30 min after surgery and the last at 24 h before sacrifice, NO agents, L-NAME 5 mg/kg (LN) or L-arginine 100 mg/kg (LA), were applied intraperitoneally, given alone, or combined, as well as combined with BPC 157 regimens, per-oral or intraperitoneal. Controls (C) received drinking water (12 mL/rat/day) or saline 5 mL/kg/day intraperitoneally. * *p* < 0.05, at least vs. control.

The skin (Figure 1 and Figure 2) defects failed to heal and failed to close.

**Figure 3:**
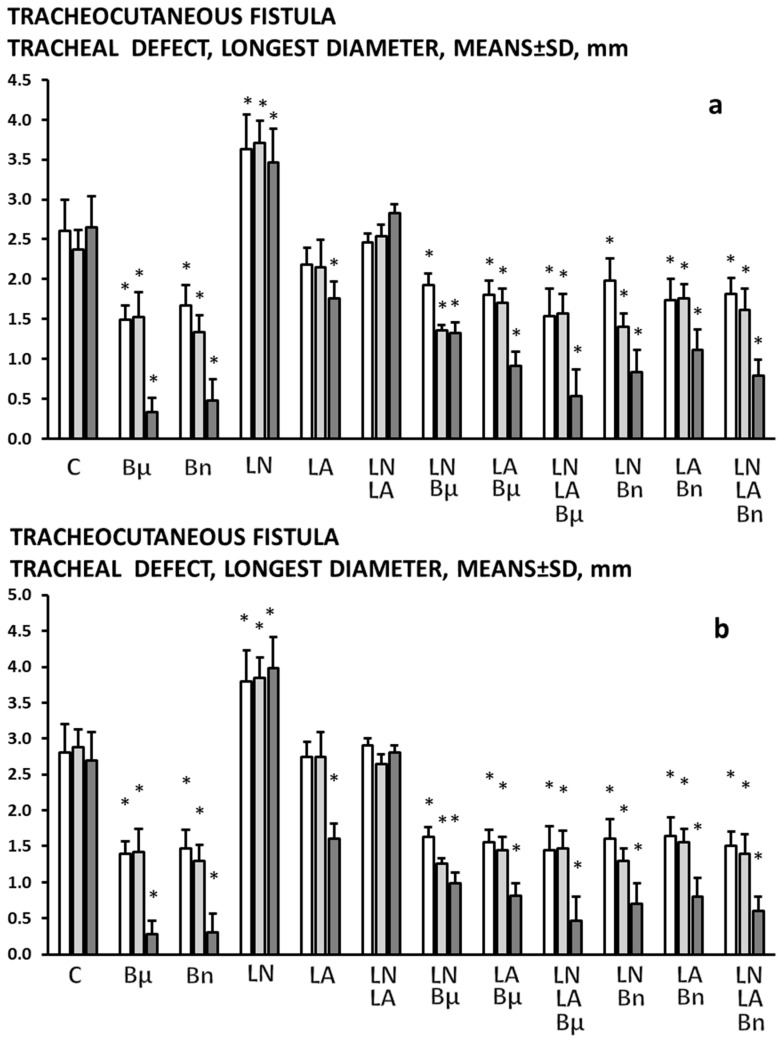
Tracheocutaneous fistula tracheal defect, longest diameter, means ± SD, mm, at post-operative day 3 (white bars), 5 (light gray bars), and 7 (dark gray bars). BPC 157 was given per-orally, in drinking water (10 μg/kg (Bμ), 10 ng/kg (Bn), i.e., 0.16 μg/mL, 0.16 ng/mL, 12 mL/rat/day) until sacrifice on the third, fifth, and seventh postoperative day (**a**) or, alternatively, 10 μg/kg, 10 ng/kg intraperitoneally, with the first application at 30 min after surgery and the last at 24 h before sacrifice (**b**). Likewise, with the first application at 30 min after surgery and the last at 24 h before sacrifice, NO agents, L-NAME 5 mg/kg (LN) or L-arginine 100 mg/kg (LA), were applied intraperitoneally, given alone, or combined, as well as combined with BPC 157 regimens, per-oral or intraperitoneal. Controls (C) received drinking water (12 mL/rat/day) or saline 5 mL/kg/day intraperitoneally. * *p* < 0.05, at least vs. control.

The tracheal (Figure 1 and Figure 3) defects failed to heal and failed to close.

**Figure 4:**
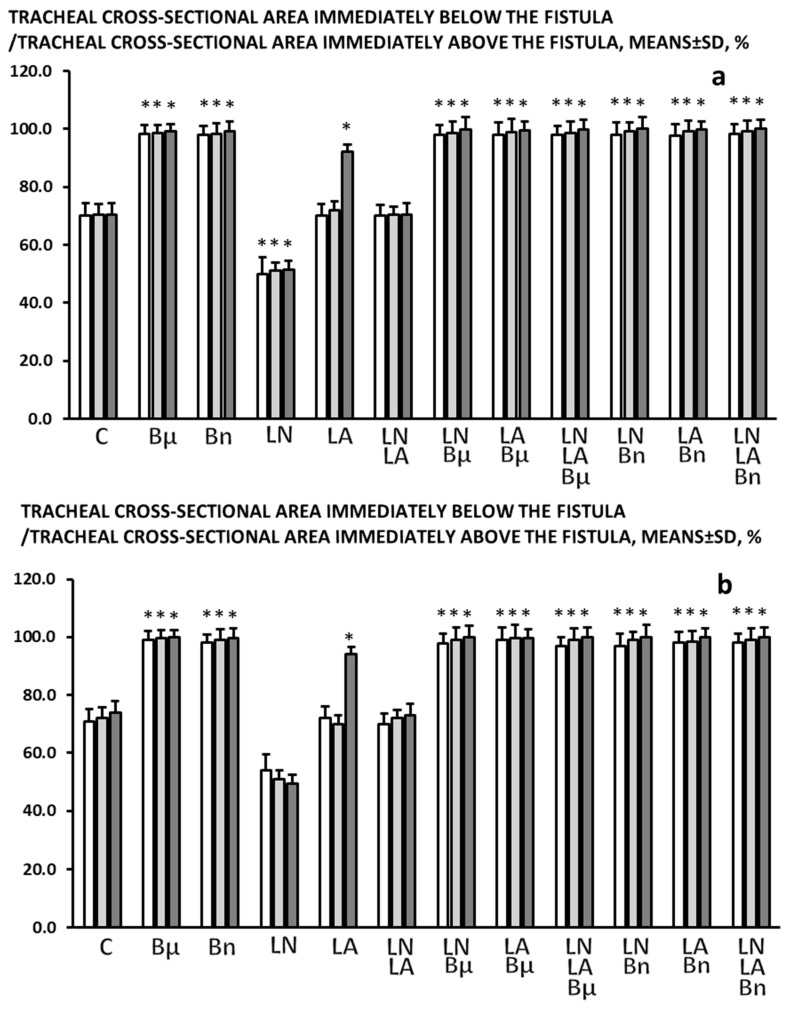
Tracheocutaneous fistula shrinking, tracheal cross-sectional area, immediately below the fistula/immediately above the fistula, means ± SD, %, at post-operative day 3 (white bars), 5 (light gray bars), and 7 (dark gray bars). BPC 157 was given per-orally, in drinking water (10 μg/kg (Bμ), 10 ng/kg (Bn), i.e., 0.16 μg/mL, 0.16 ng/mL, 12 mL/rat/day) until sacrifice on the third, fifth, and seventh postoperative day (**a**) or, alternatively, 10 μg/kg, 10 ng/kg intraperitoneally, with the first application at 30 min after surgery and the last at 24 h before sacrifice (**b**). Likewise, with the first application at 30 min after surgery and the last at 24 h before sacrifice, NO agents, L-NAME 5 mg/kg (LN) or L-arginine 100 mg/kg (LA), were applied intraperitoneally, given alone, or combined, as well as combined with BPC 157 regimens, per-oral or intraperitoneal. Controls (C) received drinking water (12 mL/rat/day) or saline 5 mL/kg/day intraperitoneally. * *p* < 0.05, at least vs. control.

There was tracheal shrinking below the fistula (Figure 4).

**Figure 5:**
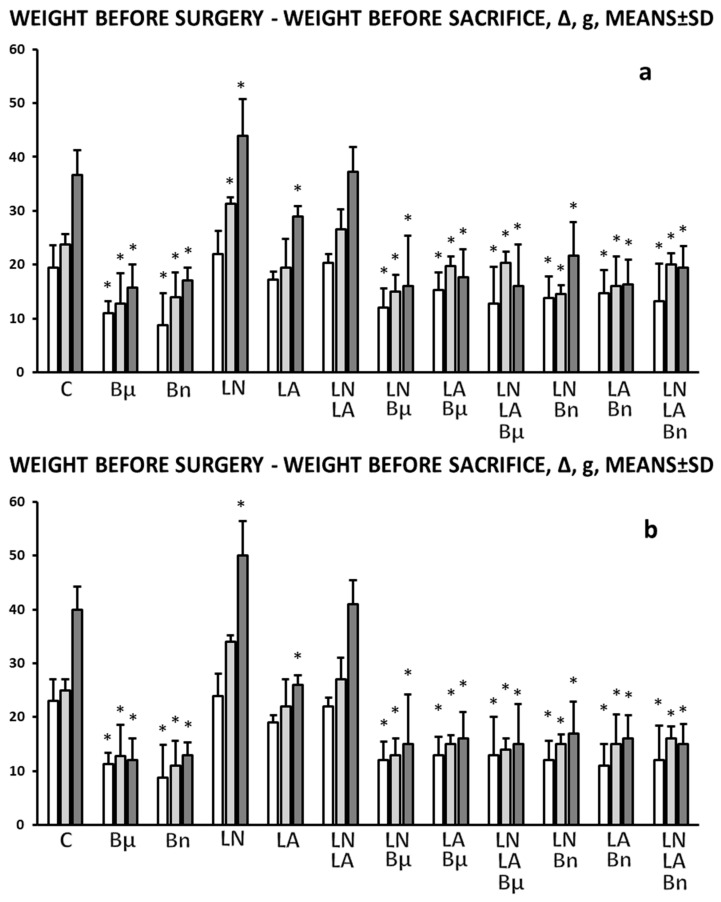
Tracheocutaneous fistula induced weight loss, weight before surgery and weight before sacrifice, Δ, means ± SD, g, at post-operative day 3 (white bars), 5 (light gray bars), and 7 (dark gray bars). BPC 157 was given per-orally, in drinking water (10 μg/kg (Bμ), 10 ng/kg (Bn), i.e., 0.16 μg/mL, 0.16 ng/mL, 12 mL/rat/day) until sacrifice on the third, fifth, and seventh postoperative day (**a**) or, alternatively, 10 μg/kg, 10 ng/kg intraperitoneally, with the first application at 30 min after surgery and the last at 24 h before sacrifice (**b**). Likewise, with the first application at 30 min after surgery and the last at 24 h before sacrifice, NO agents, L-NAME 5 mg/kg (LN) or L-arginine 100 mg/kg (LA), were applied intraperitoneally, given alone, or combined, as well as combined with BPC 157 regimens, per-oral or intraperitoneal. Controls (C) received drinking water (12 mL/rat/day) or saline 5 mL/kg/day intraperitoneally. * *p* < 0.05, at least vs. control.

There was continuous weight loss (Figure 5).

**Figure 6:**
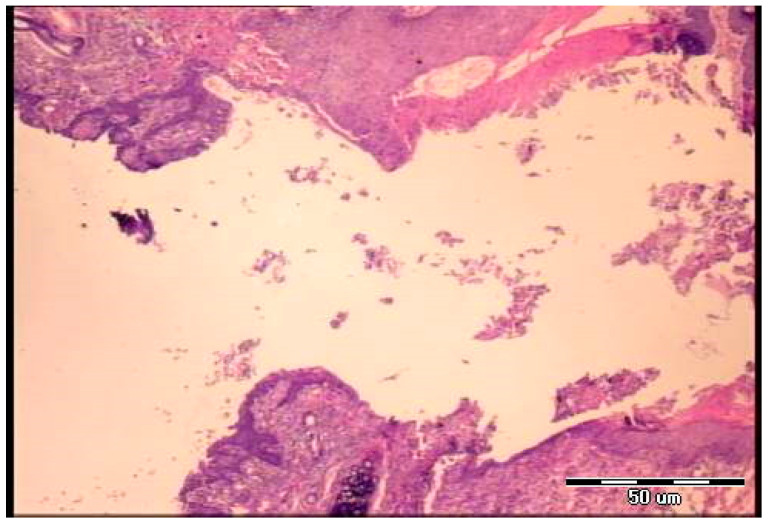
Histological presentation of the specimen, control group, fifth postoperative day (H&E, objective ×4). The fistulous tract is open, partly filled with loose edematous granulation tissue containing abundant fibrin deposits, while in another part epithelial cells are seen overgrowing the connective structurally altered portion of the tract wall.

**Figure 7:**
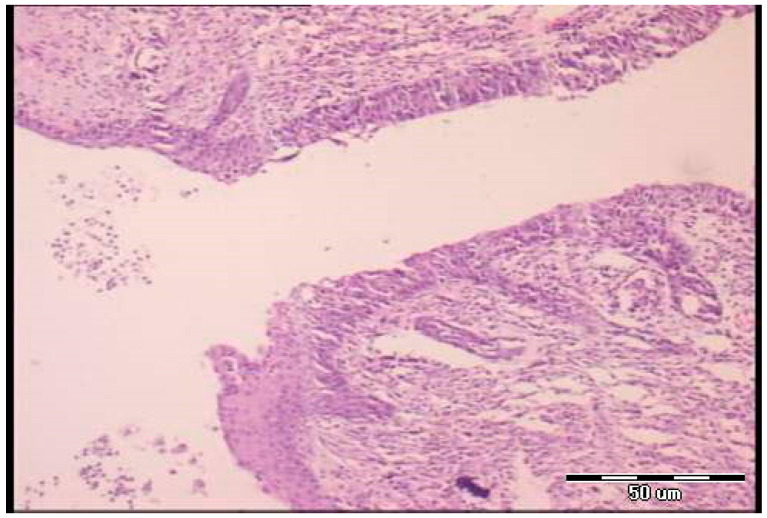
Histological presentation of the specimen, control group, seventh postoperative day (H&E staining, objective ×4). The fistulous tract is well formed and wide, lined by two types of epithelia (cutaneous and tracheal sides), with areas of granulation tissue present beneath the epithelial layer.

Microscopy demonstrated the fistulous tract to be open, partly filled with loose edematous granulation tissue containing abundant fibrin deposits, while in another part, epithelial cells are seen overgrowing the connective structurally altered portion of the tract wall (post-operative day 5). At the end, the fistulous tract is well-formed and wide, lined by two types of epithelia (cutaneous and tracheal sides), with areas of granulation tissue present beneath the epithelial layer (post-operative day 7) (Figure 6 and Figure 7). Notably, animals could not survive after the seventh day.

### 2.2. BPC 157

**Figure 8:**
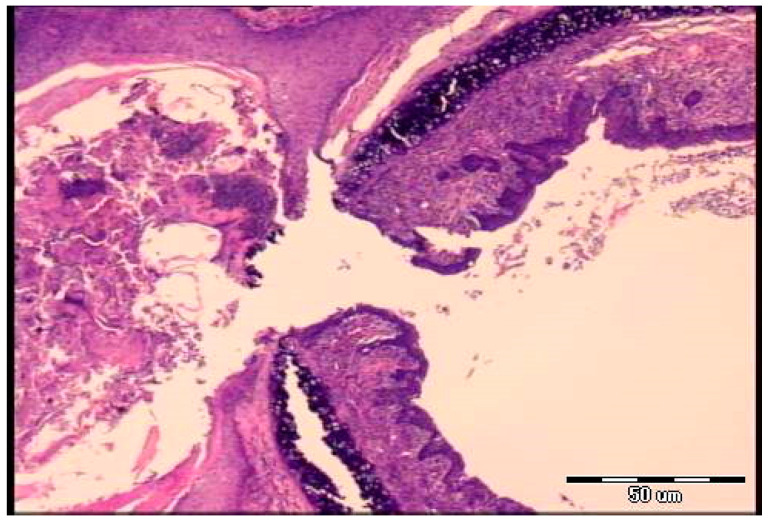
Histological presentation of the specimen, BPC 157 group, fifth postoperative day (H&E staining, objective ×10). Areas of cellular granulation tissue are observed, with a relatively scant remnant of the fistulous tract.

**Figure 9:**
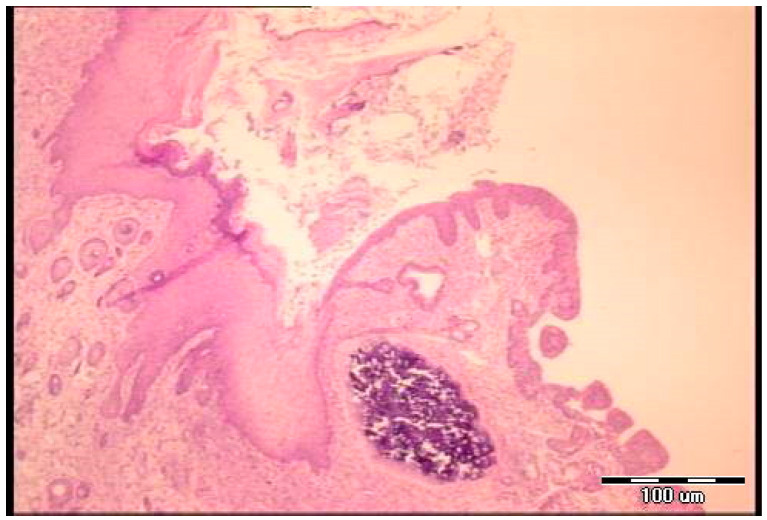
Histological appearance of the specimen, BPC group, seventh postoperative day (H&E staining, objective ×4). In treated animals, the fistulous tract is closed by granulation tissue, with areas showing epithelialization of the fistula opening.

In all BPC 157-treated rats (both µg-regimen and ng-regimen, given in drinking water or intraperitoneally) (Table 1 and Table 2, Figure 1, Figure 2, Figure 3, Figure 4 and Figure 5), there is a marked recovery of the fistula course, healing of defects, skin and trachea, as assessed by macroscopic, microscopic, and clinical observation. This was along with avoiding observable clinical signs consistent with respiratory distress from the beginning and avoidance of any sign of “heaving abdomen” presentation. The BPC 157-treated rats displayed regular breathing movements, with smooth and synchronous chest and abdominal excursions, without overt clinical signs of respiratory distress. Consistently, the weight loss was markedly reduced. There was no tracheal shrinking below the fistula. Both defects started to heal in a short period of time, more than 50% of the fistula surface was closed on the third post-operative day and almost completely closed on the seventh post-operative day. With a relatively scant remnant of the fistulous tract (post-operative day 5) (Figure 8), the fistulous tract was closed by granulation tissue, with areas showing epithelialization of the fistula opening (post-operative day 7) (Figure 9).

### 2.3. L-NAME

An already detrimental course of the tracheocutaneous fistulas further deteriorated (Table 1 and Table 2, Figure 1, Figure 2, Figure 3, Figure 4 and Figure 5). Shortly after the formation of the tracheocutaneous fistula, the L-NAME-treated rats exhibited marked clinical signs of respiratory distress due to air leakage, including pronounced abdominal excursions (“heaving abdomen”), flared nostrils, open-mouth breathing, and cyanosis (bluish coloration of the snout, ears, and extremities). There was abundant secretion through the fistula and markedly reduced activity. With such a course, both the skin and tracheal defects were larger and remained open without healing, accompanied by tracheal narrowing below the fistula, and continuous increased weight loss was observed. Microscopy investigation demonstrated that the fistulous tract remained widely open, extensively filled with loose edematous granulation tissue and dense fibrin deposits, while the epithelial overgrowth was sparse, irregular, or incomplete over the structurally compromised connective tissue, indicating delayed or impaired healing.

### 2.4. L-Arginine

The otherwise persistent detrimental course of the tracheocutaneous fistulas appeared to be improved at a later interval, providing a reduced size of both skin and tracheal defect, which, however, remained open (Table 1 and Table 2, Figure 1, Figure 2, Figure 3, Figure 4 and Figure 5). The initially severe course presented similar to the controls and was later reversed. Notably, at these points, there was reduced weight loss. The tracheal shrinking was less expressed. Microscopy investigation demonstrated that the fistulous tract was reduced in size, with smaller skin and tracheal defects, yet remained partially open. The tract contained more organized granulation tissue with less edema and fibrin, and epithelial cells began to cover the connective structurally altered portions of the tract wall, indicating early but incomplete healing.

### 2.5. L-NAME+L-Arginine

With combined administration, the course of the tracheocutaneous fistula followed the presentation as in the control rats (Table 1 and Table 2, Figure 1, Figure 2, Figure 3, Figure 4 and Figure 5). Thereby, it seems that the aggravating course of L-NAME and the ameliorating course of L-arginine, when combined, oppose each other’s effects, and thereby, their effects appear to be specifically NO-related.

### 2.6. BPC 157 and NO Agents

The pronounced abdominal excursions or “heaving abdomen” during respiration, flared nostrils, and open-mouth breathing, cyanosis (bluish snout, ears, extremities), abundant secretion through the fistula, and very reduced activity, defects in skin and trachea failing to heal, and prominent weight loss did not occur with combined administration. With BPC 157 and NO agents, the course of the tracheocutaneous fistula followed the presentation as in the BPC 157 rats (Table 1 and Table 2, Figure 1, Figure 2, Figure 3, Figure 4 and Figure 5). Thereby, it seems that the aggravating course of L-NAME and the ameliorating course of L-arginine, alone or combined, were all reversed by the co-administration of BPC 157, providing a course like that in the BPC 157-treated rats.

### 2.7. Oxidative Stress

**Figure 10:**
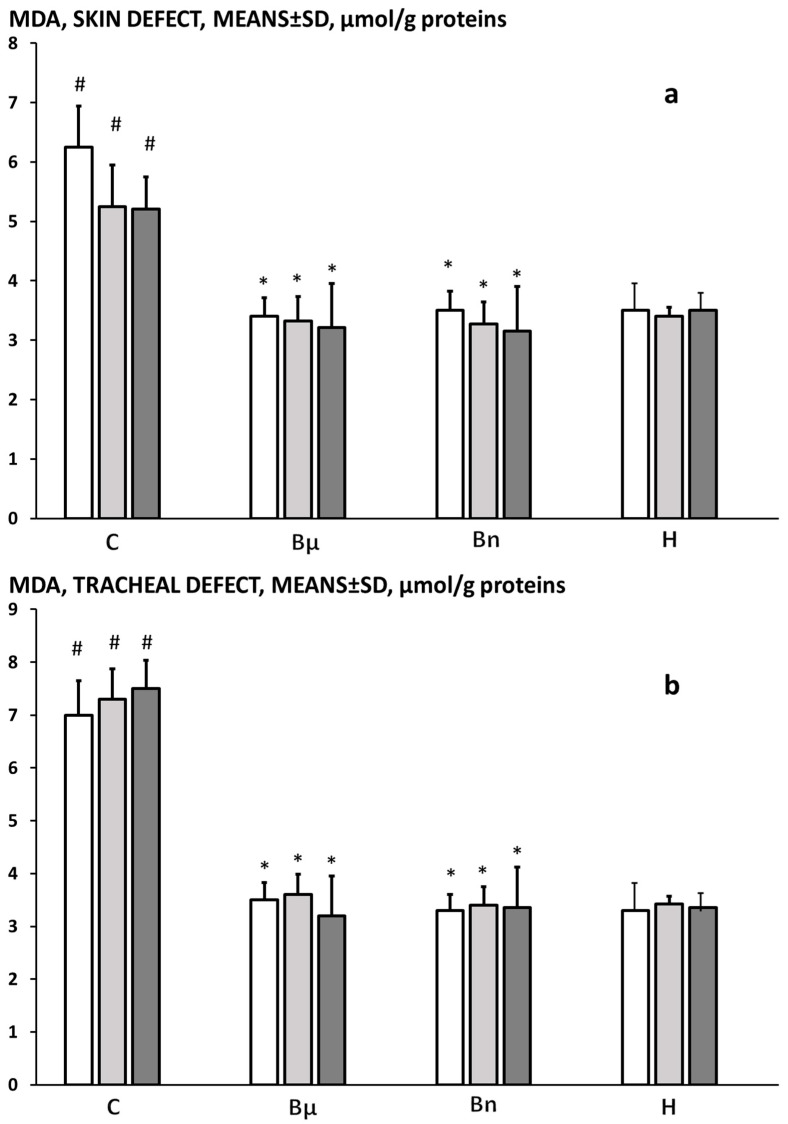
Tracheocutaneous fistula, MDA levels in skin (**a**) and tracheal (**b**) defects, means ± SD, μmol/g proteins. Presentation at post-operative day 3 (white bars), 5 (light gray bars), and 7 (dark gray bars) in healthy rats (H) and in tracheocutaneous fistula rats that received BPC 157 given per-orally, in drinking water (10 μg/kg (Bμ), 10 ng/kg (Bn), i.e., 0.16 μg/mL, 0.16 ng/mL, 12 mL/rat/day) until sacrifice or drinking water (12 mL/rat/day) (controls) (C). * *p* < 0.05, at least vs. control. # *p* < 0.05, at least vs. healthy.

At 3, 5, and 7 days post-injury, the MDA levels were markedly increased in tracheocutaneous fistula rats over the healthy values, showing prominent oxidative stress in the skin and tracheal defects. These increased MDA levels were counteracted and normalized to the healthy values in both the skin and tracheal tissues by BPC 157 therapy (Figure 10).

### 2.8. NO Levels

**Figure 11:**
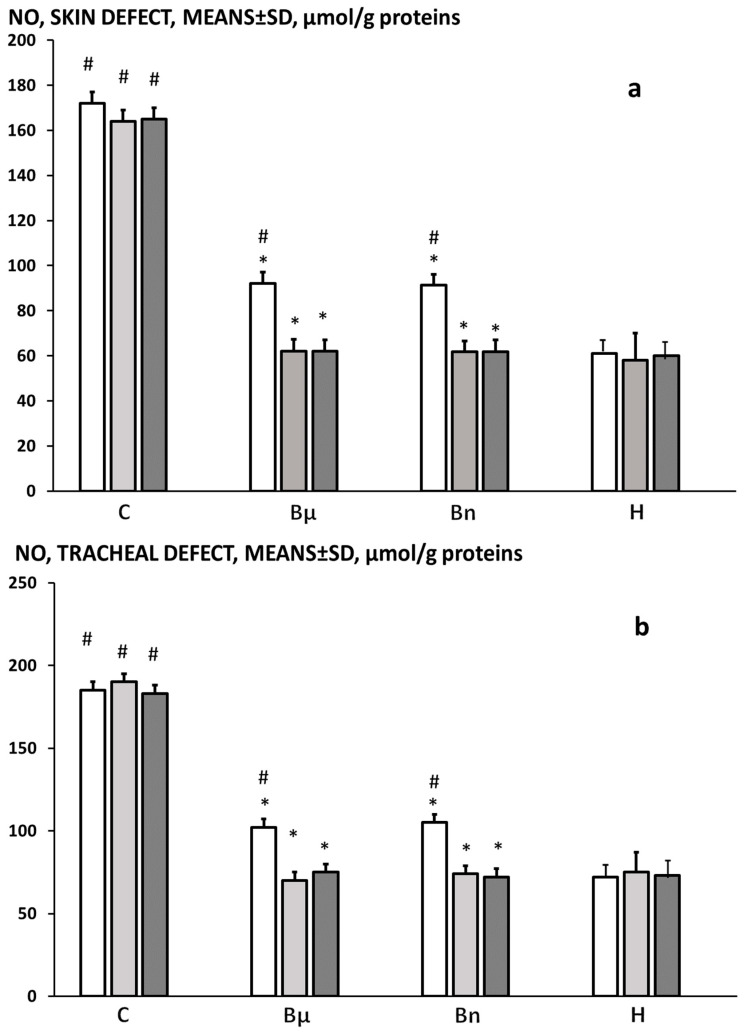
Tracheocutaneous fistula, NO levels in skin (**a**) and tracheal (**b**) defects, means ± SD, μmol/g proteins. Presentation at post-operative day 3 (white bars), 5 (light gray bars), and 7 (dark gray bars) in healthy rats (H) and in tracheocutaneous fistula rats that received BPC 157 given per-orally, in drinking water (10 μg/kg (Bμ), 10 ng/kg (Bn), i.e., 0.16 μg/mL, 0.16 ng/mL, 12 mL/rat/day) until sacrifice or drinking water (12 mL/rat/day) (controls) (C). * *p* < 0.05, at least vs. control. # *p* < 0.05, at least vs. healthy.

At 3, 5, and 7 days post-injury, likely as a part of the perilous course regularly occurring in tracheocutaneous fistula rats, the NO levels were markedly increased in the skin and tracheal defects over the healthy values. BPC 157 therapy counteracted the increased NO levels leading to the reversal to the healthy values in both skin and tracheal tissues (Figure 11).

In summary, this study (total number of 270 rats) revealed the interplay resulting with fistula course-induced healing failure (i.e., tracheocutaneous fistula → air leak → compensatory diaphragmatic/abdominal “heaving”, as a highlight of severe clinically observable respiratory distress, fatality of tracheocutaneous fistula, dysfunctional NO system, increased MDA levels, and increased NO levels in skin and tracheal defects). These were all rescued by BPC 157 therapy. There was a marked recovery of the fistula course and defects (skin and trachea), macro/microscopically. These corresponded to a marked clinical recovery (i.e., the resolution of pronounced abdominal excursions, a reduction in observable respiratory distress signs, and reduced weight loss), no detrimental chain of events, tracheocutaneous fistula → air leak → compensatory diaphragmatic/abdominal “heaving”. This reveals the success of BPC 157 therapy over a triple NO-agent design. This simultaneously captured inhibition (aggravation, L-NAME), overstimulation (L-arginine, amelioration), and immobilization of the NO system (L-NAME+L-arginine, opposing each other’s effect) and reveals the NO-system relation. The increased MDA levels and increased NO levels in skin and tracheal defects were both normalized.

## 3. Discussion

BPC 157 resolves the tracheocutaneous fistula—conceptualized as a particular NO-system failure. Concomitantly, it attenuates the associated cascade of respiratory distress manifestations. The success of BPC 157 therapy includes the closure of the tracheocutaneous fistula (i.e., marked recovery of the fistula course and defects (skin and trachea), macro/microscopically). This occurs along with marked clinical recovery (i.e., heavy abdomen, severe dyspnea, weight loss), no detrimental chain of events: tracheocutaneous fistula **→** air leak **→** compensatory diaphragmatic/abdominal “heaving”. Noteworthy for tracheocutaneous fistula rats, this is the first description. Nevertheless, for the definitive evidence needed to be fully functional and mechanistic, there should be direct respiratory measurements, which were not included in the present study. However, the presented effects are consistent and likely closely interrelated. In addition, considering the reduced weight loss in tracheocutaneous fistula rats, as a more general effect, BPC 157 therapy counteracted weight loss in other fistula studies (i.e., esophagocutaneous fistulas [42]). This occurs also both perorally and parenterally [78] in short-bowel rats, with weight gain above preoperative values. Likewise, in mice bearing C26 colon carcinoma, BPC 157 counteracted severe muscle cachexia and weight loss, improving anabolic pathways while counteracting catabolic pathways, and most importantly, prolonging survival [79].

Additionally, BPC 157 therapy dominates the triple NO-agent paradigm, and this effect is reproducible using both per-oral and intraperitoneal routes [6,7,8,9,10,11,12,13,14,15]. With potential clinical feasibility, BPC 157 works by two routes (in drinking water, per-orally, intraperitoneally; alone and per-orally, intraperitoneally, with NO agents given intraperitoneally). A tracheocutaneous fistula behaves as a distinct NO-system disturbance inhibition (aggravation, L-NAME), overstimulation (L-arginine, amelioration), and immobilization of the NO system (L-NAME+L-arginine, opposing each other’s effect, NO-related), and BPC 157 resolves the tracheocutaneous fistula in all relevant NO states. As emphasized, these would have considerable translational significance, given all the experiments carried out [6]. Accordingly, in tracheocutaneous fistulas, the NO level in tissue was increased, and the MDA was increased, as a perilous course that was initiated [6], and this was regularly normalized through BPC 157 administration.

Evidently, BPC 157 rapidly promoted tracheal wall healing and was associated with the restoration of coordinated thoracoabdominal breathing movements on clinical observation. BPC 157-treated rats displayed regular breathing movements, with smooth and synchronous chest and abdominal excursions. Preceding an imminent fatality, in tracheocutaneous fistula rats, the particular healing amid the tracheal stricture below the injury did not develop. In addition, it can be that the healing proceeds from the outside inward; during this process, the closure of the skin lesion contributes to filling the tracheal defect, ultimately promoting the closure of the fistula as a whole. The closure of the skin lesion also led to a reduction in the secretions from the fistula opening to the airway, resulting in an improvement in the general condition of the animals, without observable clinical signs of dyspnea or dysphagia. Otherwise, although some points were not specifically investigated, there can be a large tracheal defect, increased inspiratory effort associated with upper airway obstruction, decreased intrathoracic pressure, and a threatening chain of negative-pressure pulmonary edema. Accordingly, BPC 157 therapy was associated with the attenuation of severe lung pathological changes [72,80,81,82,83,84,85,86,87,88,89,90,91,92,93]. Illustratively, along with other beneficial effects, this was carried out in studies of severe vascular and multiorgan failure, presented as occlusion/occlusion-like syndrome [80,81,82,83,84,85,86,87,88,89,90,91,92,93]. This occurred in rats with major vessel occlusion, peripherally and centrally [80,81,82] or who underwent similar noxious procedures [83,84,85,86,87] or noxious agents [88,89,90,91,92,93].

The triple NO-agent approach in tracheocutaneous rats revealed matching results in tracheocutaneous fistulas and esophagocutaneous [43], duodenocutaneous [45], and colocutaneous [46] fistulas. NO-system relations, blockade-over-activity-immobilization, L-NAME vs. L-arginine opposite relations, and L-arginine (amelioration) vs. L-NAME relations (worsening) were constant. Commonly, the opposite points combined (L-NAME+L-arginine) resulted in the control level. These matching relations (i.e., L-NAME-responsive, L-arginine-responsive, opposite, NO-related, specific) categorize tracheocutaneous fistulas and other fistulas as a particular NO-system phenomenon. As such, it can be accordingly matched with the other NO responses that most commonly appear [6,7,8,9,10,11,12,13,14,15]. Thus, exogenous application of NO-agents mimicking endogenous NO-system presentation indicates the NO system for both healing and failed healing; specifically revealing the NO-system function (healing) and the NO-system dysfunction (worsening) can be regarded as a more general pattern for NO/fistula healing. This could be a highlight for the BPC 157 effect, given the healing of both external and internal fistulas. Notably, this matching can be an important distinction and indication for mapping a specific NO-system fistula cluster presentation. Namely, in some other studies, in distinctive targets [6,7], L-NAME and L-arginine did not have opposite effects but also had parallel effects [6,7] (i.e., [94,95,96]). These parallel effects opposed each other (i.e., NO-related, specific [94]) or do not oppose each other (NO-non-related, nonspecific [95,96]).

However, the most severe fistulas are probably tracheocutaneous, given that L-NAME aggravation occurred immediately, but L-arginine amelioration occurred later than in other fistula healing [43,45,46]. Notably, considering the extent of the beneficial effects noted in tracheocutaneous fistula healing, a similar range of the beneficial effects occurs in resolving the complexity of the healing of other fistulas. As a part of BPC 157’s general effects, ascribed to its innate cytoprotection activities [6,7,8,9,10,11,12,13,14,15], a similar range of beneficial effects occurs in the counteraction of concomitant complications of the gastrointestinal fistulas included in BPC 157/NO-system research [43,45,46]. Without therapy, esophagocutaneous fistulas are lethal [43], and the mortality rate of 40% until the fourth day occurs with duodenocutaneous fistulas [45]. Most likely, given the consistent beneficial effects, BPC 157 therapy could specifically counteract the cytotoxic and damaging actions of NO, being organ-specific [6]. As mentioned, considering tracheocutaneous fistulas with BPC 157’s modulatory effect, counteraction of the increased NO and MDA levels occurred also with vessel occlusion, cirrhosis, cytostatic application, and haloperidol application (for details, see, e.g., [6]). Moreover, confronted with the increased MDA values and the decreased NO values, BPC 157 reversed the values to normal healthy values (perforation, severe ischemic/reperfusion colitis) (for details, see, e.g., [6]). Consistently, BPC 157 acts as a free radical scavenger, as previously demonstrated [97,98,99], particularly in studies of vascular occlusion and occlusion-like failure [80,81,82,83,84,85,86,87,88,89,90,91,92,93], as well as in radiation-induced injury [98]. This activity is closely linked to its role as a stabilizer of cellular junctions [99], which significantly mitigates leaky gut syndrome [99] by increasing tight junction protein ZO-1 expression and transepithelial resistance.

Finally, with all the previous data [6,7,8,9,10,11,12,13,14,15] and the consistent findings presented in this study, we conclude that several points still preclude the full understanding of tracheocutaneous fistula as a particular NO-system failure, fully responsive to BPC 157 therapy. First, there were methodological shortcomings. First, an animal model, which is an acute and surgically induced fistula, may not fully capture the conditions of patients. Likewise, the absence of direct respiratory measurement precludes taking the evidence as fully functional and mechanistic. Therefore, any references to respiratory improvement in this study should be interpreted strictly as clinical and behavioral observations and, as yet, not evidence of restored ventilatory mechanics, gas exchange, or respiratory physiology. In addition, BPC 157, as well as the NO system [19,20,21], interacts with many molecular pathways [98,99,100,101,102,103,104,105,106]. Together, these may include far more complex processes than those investigated in the present study. However, these findings align with the general cytoprotective evidence and wound healing capabilities, including the prevention of necrosis and coordinated healing across multiple tissues [6,7,8,9,10,11,12,13,14,15]. Importantly, given its per-oral efficacy, BPC 157, a stable pentadecapeptide native and stable in human gastric juice for more than 24 h, can be released into the circulation and sent to distant organs as a cytoprotective mediator [6,7,8,9,10,11,12,13,14,15]. Its presence in human adult and fetal tissues (shown by in situ hybridization and immunostaining) further supports its physiological relevance [6,7,8,9]. Together, these findings illustrate the pleiotropic organoprotective activity of BPC 157 in fistula healing, in particular [11,12]. Notably, its efficacy is achieved at microgram–nanogram doses, applied without carrier addition, via multiple routes including oral administration [6,7,8,9,10,11,12,13,14,15]. The available clinical evidence and toxicology data, showing no LD1, underscore its practical applicability [6,7,8,9,10,11,12,13,14,15].

## 4. Materials and Methods

### 4.1. Animals

The study was conducted with appropriately randomized male albino Wistar rats, 12–16 weeks of age, 280 g in body weight, who were self-breeded in the Department of Pharmacology, Faculty of Medicine, Zagreb, Croatia. The facility for animals was registered by the Veterinary Directorate (Reg. No.: HR-POK-007). Laboratory rats were acclimatized for five days and assigned identification numbers prior to allocation. Randomization was performed using a computer-generated random number sequence with block randomization to ensure equal group sizes (*n* = 6 animals per group per time point) across all treatments and postoperative intervals. The laboratory animals were housed in polycarbonate (PC) cages in conventional laboratory conditions at 20–24 °C, relative humidity of 40–70%, and a noise level of 60 dB. The cages were identified with the dates, study number, group, dose, number, and sex of each animal. Twelve-hour daylight was provided by fluorescent lighting. They received standard nutrition (pelleted feed) and fresh water by free access (ad libitum) in accordance with Good Laboratory Practice (GLP). The care of the animals was in accordance with the standard operating procedures of the facility for pharmacological animals and the European Convention for the Protection of Vertebrate Animals Used for Experimental and Other Scientific Purposes (ETS 123). This research was approved by the local Ethics Committee (case number 380-59-10106-17-100/290) and by the Directorate of Veterinary (UP/I-322-01/15-01/22). The ethical principles of the study were in accordance with the European Directive 2010/63/EU, the Act on Amendments to the Animal Protection Act (Official Gazette 37/13), the Animal Protection Act (Official Gazette 135/06), the Ordinance on the Protection of Animals Used for Scientific Purposes (Official Gazette 55/13), the recommendations of the Federation of European Laboratory Associations for Animal Science (FELASA), and the recommendations of the Ethics Committee of the Faculty of Medicine, University of Zagreb. The experiments were evaluated by an independent observer who was blinded to the treatment allocation.

A priori power analysis was performed for a representative primary outcome (tracheal defect diameter), assuming a two-sided significance level of 0.05 and a statistical power of 80%. Based on effect sizes observed in previous studies using the same experimental model, a large standardized effect size (Cohen’s d = 1.2) was assumed. Under these conditions, a minimum of 6 animals per group was required to detect a statistically significant difference between groups. Accordingly, the group size was set at *n* = 6 animals per group per time point.

### 4.2. Drugs

As described before [11,12], medication included stable gastric pentadecapeptide BPC 157 (a partial sequence of the human gastric juice protein BPC, freely soluble in water at pH 7.0 and in saline), given without carrier or peptidase inhibitor. It was prepared as a peptide with 99% (HPLC) purity (1-des-Gly peptide was the main impurity; manufactured by Diagen, Ljubljana, Slovenia, GEPPPGKPADDAGLV, M.W. 1419), as described before (for review see [6,7,8,9,10,11,12,13,14,15]). L-NAME, and L-arginine were purchased (St. Louis, MO, USA).

### 4.3. Experimental Protocol

In deeply anesthetized rats, a 4 mm diameter tracheocutaneous fistula was surgically made. An opening in the trachea was made by a horizontal incision through the elastic connective tissue of the trachea above and below the cartilaginous ring, the fifth tracheal ring, which was then partially resected with two vertical incisions. The tracheal defect, 4 mm in diameter, was then secured to the skin edges on the anterior side of the neck with four individual 5-0 sutures (Monocryl, Ethicon Inc., Johnson & Johnson, Raritan, NJ, USA). In this way, a communication between the trachea and the skin (4 mm in diameter) was created. BPC 157 was given per-orally, in drinking water (10 μg/kg, 10 ng/kg, i.e., 0.16 μg/mL, 0.16 ng/mL, 12 mL/rat/day) until sacrifice on the third, fifth, or seventh postoperative day or, alternatively, 10 μg/kg, 10 ng/kg intraperitoneally, with the first application at 30 min after surgery and the last 24 h before sacrifice. Likewise, with the first application at 30 min after surgery and the last at 24 h before sacrifice, NO agents, L-NAME 5 mg/kg or L-arginine 100 mg/kg, were applied intraperitoneally, given alone, or combined, as well as combined with BPC 157 regimens, per-oral or intraperitoneal. Controls received drinking water (12 mL/rat/day) or saline 5 mL/kg/day intraperitoneally. The experimental design comprised control groups and treatment groups receiving BPC 157 (per oral or intraperitoneal; 10 μg/kg or 10 ng/kg), L-NAME, L-arginine, or their combinations. Outcomes were assessed at postoperative days 3, 5, and 7, with 6 animals per group per time point, resulting in a balanced factorial design across all experimental conditions.

Immediately after the sacrifice on postoperative days 3, 5, and 7, the skin and tracheal defects were assessed as described before [11,12]. Briefly, a precise caliper was used to verify the final size of the defect, and the largest diameter of the skin or trachea defect was assessed (mm), photographed with a camera attached to a VMS-004 Discovery Deluxe USB microscope (Veho, New York, NY, USA), and further verified using the program ISSA (VAMSTEC Software Company, Zagreb, Croatia), and the tissue was processed for further microscopic analysis.

In separate experiments, to determine the oxidative stress and NO-level changes in rats with a tracheocutaneous fistula in skin and tracheal defect over that in healthy rats, medication was BPC 157 given per-orally, in drinking water (10 μg/kg, 10 ng/kg), and drinking water (controls) until the sacrifice on postoperative days 3, 5, or 7.

### 4.4. Trachea Shrinking

The presentation of the trachea was recorded using gross trachea specimens from rats that had just been sacrificed, with a camera attached to a VMS-004 Discovery Deluxe USB microscope (Veho, Dayton, OH, USA). The internal rim of both tracheal openings in the image was marked using ImageJ software (version 1.53 (National Institutes of Health, Bethesda, MD, USA)), and the width of the trachea was measured. The arithmetic mean of the tracheal widths was calculated immediately below the fistula and immediately above the fistula. Then, the ratio of these two lengths was calculated as (l_1_/l_2_), where l_1_ is the arithmetic mean of the tracheal width immediately below the fistula, and l_2_ is the tracheal width immediately above the fistula.

The cross-sectional area of the trachea was calculated as follows:

(1) r=l/2,

where the radius (*r*) is defined as half of the tracheal diameter (*l*),

(2) $$A=\pi \cdot r^{2},$$

representing the area of a circle (the tracheal cross section is approximated as circle),

(3) $$A=\pi \cdot {\left(\frac{l}{2}\right)}^{2}$$

obtained by substituting Equation (1) into Equation (2).

Subsequently, the ratio of the areas (*A*_1_/*A*_2_) was determined, where *A_1_* denotes the arithmetic mean of the tracheal cross-sectional area immediately below the fistula, and *A_2_* denotes the tracheal cross-sectional area immediately above the fistula.

### 4.5. Clinical Presentation, Tracheocutaneous Fistula Syndrome, Animal Weight

For presentation of the particular syndrome after tracheocutaneous fistula formation, rats were assessed for observable clinical signs associated with the fistula due to air leakage. These included pronounced abdominal excursions (“heaving abdomen”), flared nostrils, open-mouth breathing, cyanosis (bluish coloration of the snout, ears, and extremities), abundant secretion through the fistula, and markedly reduced general activity and weight. All assessments were conducted by an observer blinded to the treatment allocation.

The animals were weighed before surgery and thereafter before sacrifice. Weight loss (g) was presented as the Δ between the initial and final weight.

### 4.6. Oxidative Stress

At 3, 5, and 7 days post-injury, oxidative stress in the skin and tracheal tissue samples was assessed by quantifying the thiobarbituric acid (TBA) reactivity as MDA equivalents. To homogenize the tissue samples, trichloroacetic acid (TCA) was added, the samples were centrifuged (3000 rpm, 5 min), and the supernatants were collected. Thereafter, 1% TBA was added, and the samples were boiled (95 °C, 60 min). The tubes were kept in ice for 10 min, and the absorbance was determined at the wavelengths of 532 and 570 nm. The concentration of MDA was read from the standard calibration curve, which was plotted using 1,1,3,3′-tetra-ethoxy propane (TEP). The extent of lipid peroxidation is expressed as the concentration of MDA, using a molar extinction coefficient for MDA of 1.56 × 105 mol/L/cm. The results are expressed in nmol/mg of protein.

### 4.7. Nitric Oxide Determination

At 3, 5, and 7 days post-injury, in the skin and tracheal tissue samples, we determined the nitric oxide (NO) levels in the stomach tissue samples using the Griess reaction (Griess Reagent System, Promega, Madison, WI, USA). Sulfanilamide was added to the homogenized tissue, which was incubated, and then, N-(1-naphthyl)ethylenediamine dihydrochloride was added. The Griess reaction is based on the diazotization reaction, in which acidified nitrite reacts with diazonium ions and, in a further step, is coupled to N-(1 naphthyl) ethylenediamine dihydrochloride to form a chromophoric azo derivative. The absorbance was measured at 540 nm using a sodium nitrite solution as the standard. The NO levels are reported in µmol/mg protein. The proteins were determined using a commercial kit (BioRad Protein DR Assay Reagent Kit, BioRad Labs., Hercules, CA, USA)

### 4.8. Statistical Analysis

The statistical analyses were conducted with parametric one-way ANOVAs with post hoc Newman–Keuls tests and non-parametric Kruskal–Wallis tests with subsequent Mann–Whitney U tests to compare groups. The values are represented as the mean ± SD. Alternatively, Fisher’s exact probability test was used. The results were considered significant at *p* < 0.05.

## 5. Conclusions

As with all animal studies, the extrapolation to human conditions has inherent limitations with the resemblance of animal models to human conditions. Nevertheless, the present rat study establishes BPC 157 as the first agent shown to systemically counteract a tracheocutaneous fistula course. This effect is achieved through the direct modulation of the NO system, providing a novel methodological framework for understanding fistula healing and NO-system dysfunction. The increased MDA levels and elevated NO levels detected in skin and tracheal defects were both normalized. This counteracted an otherwise perilous course. This effect extends across different fistula types and other NO-related disorders, highlighting the pleiotropic cytoprotective actions of BPC 157.

Along with its stability in gastric juice, oral efficacy, physiological presence in human tissues, and documented safety profile, these findings uniquely position BPC 157 as a promising candidate for clinical translation in the treatment of otherwise intractable fistulas and complex healing disturbances. Furthermore, the triple NO-agent approach enables the simultaneous investigation of NO inhibition (L-NAME), NO overactivity (L-arginine), and NO-system immobilization (L-NAME+L-arginine), representing a methodologically robust and previously unexplored strategy in tracheocutaneous fistula research. This approach provides a comprehensive mapping of tracheocutaneous fistulas as a distinct NO-system-related healing disorder.

These findings should be interpreted with consideration of both limitations (i.e., the experimental animal model and the absence of direct respiratory measurements) and advantages (i.e., dual routes of BPC 157 administration, controlled NO-agent modulation, and assessment of NO levels and oxidative stress). Taken together, the present results identify BPC 157 as the first systemically effective therapy for tracheocutaneous fistulas. Therefore, as emphasized, with all arguments mentioned before, BPC 157 emerges as a promising candidate for clinical translation in the management of otherwise intractable fistulas and complex healing disturbances.

## Figures and Tables

**Table 1:** Number of rats with and without presentation of the particular syndrome (pronounced abdominal excursions (“heaving abdomen”), flared nostrils, open-mouth breathing, and cyanosis (bluish coloration of the snout, ears, and extremities), abundant secretion through the fistula, and markedly reduced activity and weight) after tracheocutaneous fistula formation, with marked clinical signs of respiratory distress due to air leakage. BPC 157 was given per-orally, in drinking water (10 μg/kg, 10 ng/kg, i.e., 0.16 μg/mL, 0.16 ng/mL, 12 mL/rat/day) until sacrifice on the third, fifth, and seventh postoperative day. Likewise, with the first application at 30 min after surgery and the last at 24 h before sacrifice, NO agents, L-NAME 5 mg/kg or L-arginine 100 mg/kg, were applied intraperitoneally, given alone, or combined, as well as combined with BPC 157 per-oral regimen. * p < 0.05, *at least vs. control*.

Medication After Formation of Tracheocutaneous Fistula	Number of Rats Presenting with or Without (with/Without) Pronounced Abdominal Excursions or “Heaving Abdomen” During Respiration, Flared Nostrils, and Open-Mouth Breathing, Cyanosis (Bluish Snout, Ears, Extremities), Abundant Secretion Through the Fistula, and Very Reduced Activity.		
	Post-Surgery Day 3	Post-Surgery Day 5	Post-Surgery Day 7
Control (drinking water, 12 mL/rat/day)	6/0	6/0	6/0
BPC 157 10 µg/kg/po	0/6 *	0/6 *	0/6 *
BPC 157 10 ng/kg/po	0/6 *	0/6 *	0/6 *
L-NAME 5 mg/kg ip	6/0	6/0	6/0
L-arginine 100 mg/kg ip	6/0	6/0	0/6 *
L-NAME 5 mg/kg ip L-arginine 100 mg/kg ip	6/0	6/0	6/0
BPC 157 10 µg/kg/po L-NAME 5 mg/kg ip	0/6 *	0/6 *	0/6 *
BPC 157 10 µg/kg/po L-arginine 100 mg/kg ip	0/6 *	0/6 *	0/6 *
BPC 157 10 µg/kg/po L-NAME 5 mg/kg ip L-arginine 100 mg/kg ip	0/6 *	0/6 *	0/6 *
BPC 157 10 ng/kg/po L-NAME 5 mg/kg ip	0/6 *	0/6 *	0/6 *
BPC 157 10 ng/kg/po L-arginine 100 mg/kg ip	0/6 *	0/6 *	0/6 *
BPC 157 10 ng/kg/po L-NAME 5 mg/kg ip L-arginine 100 mg/kg ip	0/6 *	0/6 *	0/6 *

**Table 2:** Number of rats with and without presentation of the particular syndrome (pronounced abdominal excursions (“heaving abdomen”), flared nostrils, open-mouth breathing, and cyanosis (bluish coloration of the snout, ears, and extremities), abundant secretion through the fistula, and markedly reduced activity and weight) after tracheocutaneous fistula formation, with marked clinical signs of respiratory distress due to air leakage. BPC 157 was 10 μg/kg, 10 ng/kg intraperitoneally, with the first application at 30 min after surgery and the last at 24 h before sacrifice. Likewise, with the first application at 30 min after surgery and the last at 24 h before sacrifice, NO agents, L-NAME 5 mg/kg or L-arginine 100 mg/kg, were applied intraperitoneally, given alone, or combined, as well as combined with the BPC 157 intraperitoneal regimen. * p < 0.05, *at least vs. control*.

Medication After Formation of Tracheocutaneous Fistula	Number of Rats Presenting with or Without (with/Without) Pronounced Abdominal Excursions or “Heaving Abdomen” During Respiration, Flared Nostrils, and Open-Mouth Breathing, Cyanosis (Bluish Snout, Ears, Extremities), Abundant Secretion Through the Fistula, and Very Reduced Activity.		
	Post-Surgery Day 3	Post-Surgery Day 5	Post-Surgery Day 7
Control (drinking water, 12 mL/rat/day)	6/0	6/0	6/0
BPC 157 10 µg/kg/ip	0/6 *	0/6 *	0/6 *
BPC 157 10 ng/kg/ip	0/6 *	0/6 *	0/6 *
L-NAME 5 mg/kg ip	6/0	6/0	6/0
L-arginine 100 mg/kg ip	6/0	6/0	0/6 *
L-NAME 5 mg/kg ip L-arginine 100 mg/kg ip	6/0	6/0	6/0
BPC 157 10 µg/kg/ip L-NAME 5 mg/kg ip	0/6 *	0/6 *	0/6 *
BPC 157 10 µg/kg/ip L-arginine 100 mg/kg ip	0/6 *	0/6 *	0/6 *
BPC 157 10 µg/kg/ip L-NAME 5 mg/kg ip L-arginine 100 mg/kg ip	0/6 *	0/6 *	0/6 *
BPC 157 10 ng/kg/io L-NAME 5 mg/kg ip	0/6 *	0/6 *	0/6 *
BPC 157 10 ng/kg/ip L-arginine 100 mg/kg ip	0/6 *	0/6 *	0/6 *
BPC 157 10 ng/kg/ip L-NAME 5 mg/kg ip L-arginine 100 mg/kg ip	0/6 *	0/6 *	0/6 *

## Data Availability

The original contributions presented in the study are included in the article. Further inquiries can be directed to the corresponding authors.
